# Deep learning enabled label-free microfluidic droplet classification for single cell functional assays

**DOI:** 10.3389/fbioe.2024.1468738

**Published:** 2024-09-18

**Authors:** Thibault Vanhoucke, Angga Perima, Lorenzo Zolfanelli, Pierre Bruhns, Matteo Broketa

**Affiliations:** ^1^ Institut Pasteur, Université Paris Cité, Institut National de la Santé et de la Recherche Médicale (INSERM), Unité Mixte de Recherche (UMR) 1222, Antibodies in Therapy and Pathology, Paris, France; ^2^ Sorbonne Université, Collège Doctoral, Paris, France; ^3^ Laboratoire de Colloides et Matériaux Divisés, École Supérieure de Physique et de Chimie Industrielles de la Ville de Paris, Université Paris Sciences et Lettres, Centre National de la Recherche Scientifique (CNRS) Unité Mixte de Recherche (UMR) 8231, Paris, France; ^4^ Paris Est Créteil University (UPEC), Assistance Publique-Hôpitaux de Paris (AP-HP), Henri Mondor Hospital, Fédération Hospitalo-Universitaire TRUE InnovaTive theRapy for immUne disordErs, Créteil, France; ^5^ Evexta Bio, Paris, France

**Keywords:** droplet-based microfluidic, convolutional neural network, image classification, deep learning, image preprocessing, Resnet 50

## Abstract

Droplet-based microfluidics techniques coupled to microscopy allow for the characterization of cells at the single-cell scale. However, such techniques generate substantial amounts of data and microscopy images that must be analyzed. Droplets on these images usually need to be classified depending on the number of cells they contain. This verification, when visually carried out by the experimenter image-per-image, is time-consuming and impractical for analysis of many assays or when an assay yields many putative droplets of interest. Machine learning models have already been developed to classify cell-containing droplets within microscopy images, but not in the context of assays in which non-cellular structures are present inside the droplet in addition to cells. Here we develop a deep learning model using the neural network ResNet-50 that can be applied to functional droplet-based microfluidic assays to classify droplets according to the number of cells they contain with >90% accuracy in a very short time. This model performs high accuracy classification of droplets containing both cells with non-cellular structures and cells alone and can accommodate several different cell types, for generalization to a broader array of droplet-based microfluidics applications.

## Introduction

Droplet-based microfluidics (DBMF) have been a breakthrough in the realm of biological research, having been applied to molecular analyses and single cell analyses, particularly in the field of immunology. DBMF assays offer significantly improved throughput and parallelization while also minimizing reagent usage by miniaturizing the traditional well-plate format. Despite the apparent improvements offered by the adaptation of existing assays into DBMF formats for single cell characterization, there is a technical limitation imposed by the nature of current droplet generation methods. The encapsulation of cells into droplets follows Poisson distribution patterns of in-droplet cell numbers and is an inherently heterogeneous process (Collins et al., 2015; Moon et al., 2011). For DBMF methods to achieve true single-cell resolution, final analysis of single cells may be achieved by a high cellular dilution factor prior to encapsulation or a means of sorting for single-cell/-object droplets following encapsulation. Various methods have implemented single cell sorting either as a physical droplet isolation step using dielectrophoresis, a piezoelectric actuator, or a solenoid actuator (Dortaj et al., 2024; Murphy et al., 2018; Nakamura et al., 2024; Welch et al., 2024) or alternatively as an analytical filter applied during data analysis (Sesen and Whyte, 2020; Srikanth et al., 2021); both approaches have recently begun to be driven using single cell content classifications made by machine learning algorithms.

Machine learning has seen considerable growth in recent years regarding its technical capabilities as well as its ease of applicability and robustness, reshaping profoundly various technological sectors including autonomous vehicles (Qiu et al., 2024; Ušinskis et al., 2024), natural language processing (Vaswani et al., 2017) or automated medical image analysis (Liu et al., 2022; Qiu et al., 2024). Due to their successful application in image classification tasks and increased accessibility, Convolutional Neural Network (CNN) architectures have paved the way for the exploitation of deep learning methods in biotechnology (Kusumoto and Yuasa, 2019; Samukhina et al., 2021; Sultana et al., 2018). CNNs can directly extract information from input images: they are implemented as a deep series of convolutional layers, where at each convolutional layer a sliding filter is applied to the input image to produce an activation map of the detected features. Layer after layer this information is progressively pooled and simplified to obtain, at the last output layer, a categorical classification for the input image. The fact that all the model parameters can be learned directly from the data, using a set of manually classified training images, simplifies the application and exploitation of these architectures.

Various machine learning models have been developed to address the needs of those working on microfluidics (McIntyre et al., 2022; Riordon et al., 2019). Microfluidic devices and the experimental procedures they are employed under are now able to be artificially designed by machine learning, as well as the analysis of droplets for classification and sorting (Srikanth et al., 2021). Deep learning methods offer considerable improvement when handling structured data types, namely, images within the context of DBMF. The availability of numerous publicly available deep learning solutions offers researchers means to automatedly identify droplets containing a single cell and extract biophysical feature data, increasing the throughput and breadth of analyses (Soldati, Ben, et al., 2018). CNNs have been used for high throughput sorting of droplets according to the number of objects they contain with the added functionality to distinguish mixtures of in-droplet objects, such as a cell and bead mixture (Anagnostidis et al., 2020). Another method of droplet classification recently demonstrated the classification of various cell types despite having visually similar presentations when manually analyzed (Chen et al., 2016).

Parallel to the application of machine learning to DBMF, another emerging field of DBMF has been in-droplet secretion assays for single cell functional characterization (Broketa and Bruhns, 2022; Bucheli et al., 2021). These techniques often utilize microbeads (Konry et al., 2011), rods (Wei et al., 2019), or nanoparticles (Eyer et al., 2017) as surfaces for assay reactions or reagent and analyte capture. The detection of secreted analytes by surface capture is the basis for several widely used techniques in biomedical research (Bucheli et al., 2021; Janetzki et al., 2014), namely, ELISA and ELISPOT or FLISA and FLUOROSPOT depending on whether detection is enzyme or fluorophore-based, respectively. Single cell DBMF secretion assays will likely become increasingly common given the increased throughput and parallelization afforded by such techniques, as has been seen with DBMF techniques for single cell molecular characterization compared to their well plate-based predecessors (Sánchez Barea et al., 2019). The additional data that may be extracted from the images gathered during DBMF techniques, such as cell morphology or motility, also allows one to address unexplored aspects of functional heterogeneity.

The presence of non-cellular structures within the droplet represents an additional challenge during analyses where cellular features require identification. Cell detection and quantification during image analyses has been extensively addressed and numerous informatic packages are available to incorporate into image analysis pipelines (Buggenthin et al., 2013; Maddalena et al., 2022). Cell detection is commonly performed using fluorescent surface markers when possible or detection in the brightfield channel when label-free conditions are necessary. However, during DBMF secretion assays the included analyte capture surface (beads or rods) will introduce its own fluorescent signals or brightfield distortions. DropMap (Eyer et al., 2017) is a fluorescence relocation-based immunoassay using magnetically aligned nanobeads in droplets that we previously applied to measure antibody affinity and secretion rate in the context of autoimmune disorder (Canales-Herrerias et al., 2022) or vaccination (Broketa et al., 2023). Analyses of such single cell DBMF secretion assays that use non-cellular capture surfaces have largely been limited by a need for manual droplet verification or experimental restrictions. A requirement for manual droplet verification by the experimenter represents a significant analysis bottleneck, is less robust when applied over larger datasets, and reduces comparability of results between studies.

In this article we describe a deep learning solution using ResNet-50 for label-free droplet classification that is resistant to vertical non-cellular structures within the droplet. We used a droplet image dataset from a previous publication (Broketa et al., 2023) using the DropMap DBMF system. ResNet-50 models have been showed to be highly performant to classify droplets containing cells only (Soldati, Ben, et al., 2018). However, when applied directly to minimally preprocessed images this CNN poorly performed with droplets containing non-cellular structures that could partially mask the cells. To compensate for this drop in performance, we designed a pre-processing method to remove information irrelevant to classification (for instance the non-cellular structures) while emphasizing meaningful information (in this case the cells). Thanks to this pre-processing, our trained model is capable of classifying droplets as containing zero, one, or multiple cells with accuracy scores above 90%, therefore allowing for automated analyses of DropMap assays. This work addresses a gap in the current capabilities of machine learning-enabled image analyses to accommodate non-cellular structures that can obscure or mimic cells.

## Methods

### Wafer production

The silicone wafer for microfluidic device production was fabricated using soft lithography. Wafers were spin-coated with SU-8 2035 (MicroChem) at 3,000 rpm for 30 s, then heated for pre-baking at 65°C for 5 min followed by heating at 95°C for 3 min. Afterwards, wafers were exposed to UV light (365 nm) for 16 s using MJB-4 (SUSS MicroTec). Subsequently, wafers were heated for post-baking at 65°C for 3 min and at 95°C for 1 min. Wafers then underwent development to remove the SU-8 resin residue for 2 min at 100 rpm. Thickness was measured using a DEKTAK profilometer (Bruker) to ensure ∼40 µm of thickness.

### Microfluidic chip production

A mix of Sylgard 184 (Dow, 1317318) prepared at a 10:1 mix ratio was poured onto the wafer covered on its rear by round aluminum foil to create a mold. Subsequently, a desiccator was used to remove bubbles and the wafer was incubated in an oven at 65°C for 2 h. Afterwards, the foil mold and wafer were removed, appropriate holes were punched with a 0.75 mm biopsy puncher, and the cured PDMS was plasma bonded with glass slide 75 mm × 50 mm (Corning, 2947-75 × 50). The microfluidic chips were then silanized using 5% v/v solution of 1H, 1H, 2H, 2H-Perfluorododecyltrichlorosilane (Sigma, 729965) in HFE7500 oil (3M, B40045191).

### Droplet formation

Water-in-oil droplets were produced by mixing two aqueous phases. Phase 1 consisted of a cell suspension (30e6 cells/mL) and phase 2 consisted of assay buffer with or without paramagnetic nanobeads as indicated. Both phases were injected with a flow rate of 70 μL/h. The oil phase consisted of 2% v/v solution of Perfluorosurfactant (RAN-Biotechnologies, 008-FluoroSurfactant) in HFE 7500 oil, with a flow rate of 600 μL/h to obtain a final droplet volume of ∼40 pL.

### Computing

Computation was done with a Windows 10, 64-bit operating system using an AMD 3990X 4.3 GHz processor with 64 GB RAM and GeForce RTX 3080. All image data was stored and read from a local M.2 NVME SSD drive. Scripts were written in Python 3.7.11 utilizing the Tensorflow (Abadi et al., 2016) 2.5.0 library. Scripts used in this work are available as ESI.

### Image acquisition

An inverted microscope with a motorized stage (Nikon, Ti2-Eclipse) was used to acquire droplet images with a high-speed CMOS camera (Orca Flash 4.0, Hamamatsu) at room temperature. Images were acquired through a × 10 objective (NA 0.45). The whole of the droplet chamber was acquired as an array of 9 × 9 individual images stitched together to form a single image. Image acquisition occurred every 7.5 min over a total period of 37.5 min (6 measurements in total). Duplicates were systematically acquired for every sample, with each replicate consisting of the filling of the DropMap 2D chamber with a novel droplet population. Images were stored in the nd2 file format.

### Datasets

Droplets were identified as previously described (Broketa et al., 2023) based on circle detection by Hough transform. Droplet images for model training were taken from the brightfield channel of the original nd2 format image, centered on the droplet, and cropped to the diameter of the droplet. Images were saved in the tif file format. Droplet images were randomly selected without prior measurement or filtering based on droplet contents. Images for the creation of the validation datasets were selected amongst those images not included in the training dataset. Droplet images were visually classified by the authors according to the number of cells inside of the droplets.

### Image preprocessing

The droplet image was either loaded from a tif file into a Python 3.7.11 environment using scikit-image v0.19.2 during training or cropped directly from the nd2 file according to measured position and diameter. Then, the image undergoes either a minimal or a more sophisticated preprocessing, where indicated. In the latter, the image is convolved using a Prewitt operator kernel, with the convolve2d function from the scipy.signal package. Image contours are then highlighted using a bisigmoidal filter based on Equation 1:

$$\text{final pixel value}=24000\times \left(\frac{1}{1+e^{\alpha_{1}}}+\frac{1}{1+e^{-\alpha_{2}}}-\frac{1}{2}\right)$$ (1)

where $\alpha_{1}=\min\left(\text{initial pixel value}-\frac{\text{image}\;\text{minimal}\;\text{pixel}\;\text{value}+\text{image}\;\text{mean}\;\text{pixel}\;\text{value}}{600},700\right)$

and $\alpha_{2}=\min\left(\text{initial pixel value}-\frac{\text{image}\;\text{maximal}\;\text{pixel}\;\text{value}+\text{image}\;\text{mean}\;\text{pixel}\;\text{value}}{600},700\right)$

The image is resized to dimensions of 55 × 55 pixels using the resize function without anti-aliasing from skimage.transform package. The pixel values are then normalized using Equation 2:

$$\text{final pixel value}=\frac{\text{initial}\;\text{pixel}\;\text{value}-\text{image}\;\text{minimal}\;\text{pixel}\;\text{value}}{\text{image}\;\text{maximal}\;\text{pixel}\;\text{value}-\text{image}\;\text{minimal}\;\text{pixel}\;\text{value}}\times 65535$$ (2)

A circular mask with a 5-pixel radius reduction compared to the image half-width is applied to the image to occlude the area outside of the droplet. When minimally preprocessed, the image is resized to dimensions of 55 × 55 pixels using the resize function without anti-aliasing from skimage. transform package. Pixel values were then normalized using (Equation 2). Images are finally all passed to the model as an array of (n, 55, 55) with n representing the number of images to be classified.

### Convolutional neural network implementation

Droplet images were separated into three classes: “Empty/0”, if the droplet contained no cell, “Single/1”, if 1 cell is within the droplet, and “Multiple/2”, if two or more cells are inside the droplet.

The Keras module of the TensorFlow v2.5 library in Python 3.7.11 was used to define a ResNet-50 model. Images were resized to a uniform dimension of 55 × 55 pixels. Categorical cross-entropy was used to compute loss during training. The learning rate was set to 0.001 during training. Hyper-parameters were incrementally increased to determine optimal values. Final model training was performed using 6 epochs and a batch size of 160. The training-set contained 95% of all labeled images with the remaining 5% used for the validation-set.

The model used to classify droplets containing cells only was trained with 12 epochs, batches of 30 images and 2,500 training images for each class.

### Computer-vision-based methods implementation

Scripts for conventional object recognition were run in Matlab 2021a. Rosenfeld method was performed using the script previously published (Rosenfeld et al., 2005), run with a “dilatation” parameter of 15 instead of 7, to allow for the unique and unintended nature of our dataset.

Morphological shape algorithm method is similar with the method published by Buggenthin and colleagues (Buggenthin et al., 2013). On this method, we sequentially detect the edge, add a circular mask to the image, create linear structuring elements, dilate the image, fill the holes, erode the images and find the image centroid. Number of cells was determined based on how many image centroid were calculated.

## Results

**FIGURE 1:**
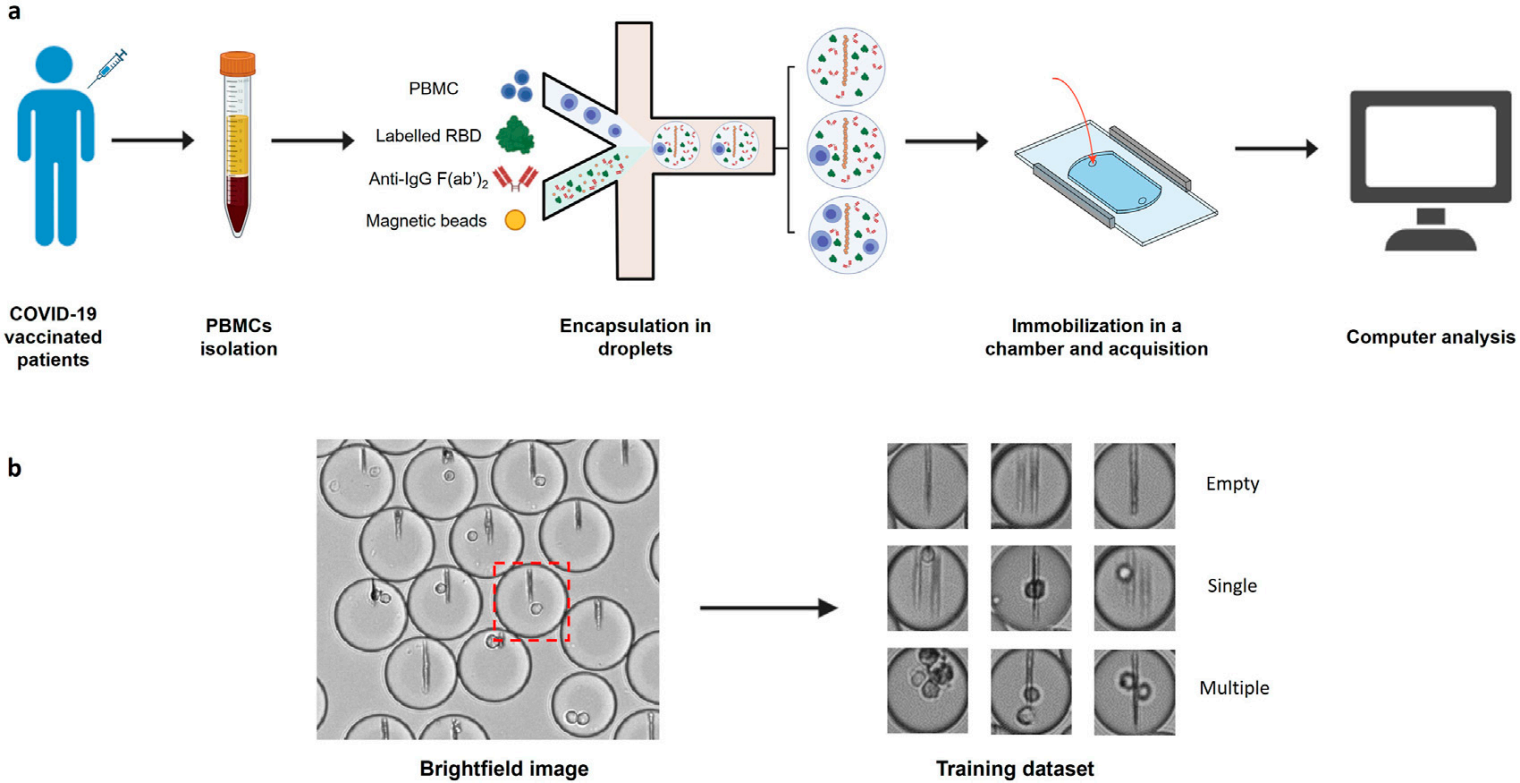
Image datasets construction. **(A)** PBMCs were isolated from COVID-19 vaccinated patient blood and injected into a microfluidic chip together with a mixture of anti-human-κ chain-VHH-coated paramagnetic nanobeads, fluorescent antigen (recombinant SARS-CoV-2 Spike Receptor Binding Domain) and fluorescent anti-human IgG F(ab’)_2_. The mix of cells and reagents was encapsulated in droplets using a flow focusing technique. The produced droplets were introduced and immobilized in an observation chamber. A magnetic field generated by two magnets was applied to the chamber so that the nanobeads form a vertical line. **(B)** Brightfield images of the chamber were acquired with an optical microscope. Images of each droplet were cropped (red square) and manually sorted into 3 populations (empty droplets, single cell droplets, and multiple cell droplets).

In this work, we used an image dataset from previously published experiments (Figure 1A) (Broketa et al., 2023). In these experiments PBMCs were isolated from COVID-19 vaccinated patients’ blood and encapsulated into ∼40 pL droplets together with paramagnetic nanobeads, fluorescently-labeled Receptor Binding Domain (RBD) of SARS-CoV-2 Spike protein and a fluorescent anti-human IgG F(ab’)_2_. Nanobeads are coated with an anti-human-κ-chain VHH that captures secreted antibodies in the event that an antibody-secreting cell is encapsulated within the droplet. Under a magnetic field, the beads in each droplet form a line of beads (hereafter named beadline) that acts as a physical surface for a sandwich immunoassay. The fluorescent anti-human IgG F(ab’)_2_ and fluorescent RBD allow us to quantify antibody relocation to the beadline and to identify RBD-specific plasma cells, respectively. The droplets were then immobilized in an observation chamber and images of the chamber were acquired over a period of about 40 min to monitor antibody secretion. We generated from these experiments three classifications of droplet images as either empty, containing a single cell, or containing multiple cells, through sorting of randomly selected droplet images (Figure 1B). Each classification contained 1,000 brightfield droplet images. We also generated three similar image datasets of droplets containing cells only to compare the accuracy of conventional methods in the presence or the absence of non-cellular structures (here nanobeads).

Conventional image analyses for cell detection use image processing algorithms such as morphological or thresholding-based transformation to identify objects within images and are preferred due to their relative simplicity and robustness (Gao et al., 2013; Macfarlane et al., 2021). The detection of cells from microscopy images is well established and several approaches are readily available through standard bioinformatic analysis packages (Cuevas et al., 2013; Li et al., 2008; Markovic et al., 2013). We therefore initially sought to use conventional object recognition to address the need for determining the number of cells within droplets. For each dataset we tested three common cell detection methods for their ability to classify droplets according to the number of cells they contained: Hough transform (Djekoune et al., 2017; Ioannou et al., 1999), Maximally Stable Extremal Regions (MSER) algorithm (Buggenthin et al., 2013; Zhang et al., 2022), and segmentation algorithm (Rosenfeld et al., 2005) (Supplementary Figure S1).

When classifying droplets containing cells only, all conventional methods tested reached an accuracy above 99% for empty droplets (Table 1). The accuracies for single-cell droplets was 85%, 21% and 83%, and for multiple-cells was 20%, 26% and 34%, when using Hough transform, MSER algorithm, or segmentation algorithm, respectively. When nanobeads forming a beadline were present within the droplets, the scores changed considerably, with accuracy for single-cell droplets dropping from 85% to 31% for Hough transform and from 83% to 62% for the segmentation algorithm, whereas increasing from 21% to 38% for the MSER algorithm. Overall conventional cell detection methods performed poorly to accurately quantify the number of cells in a droplet, especially when the droplet contained nanobeads, despite their proven accuracy and robustness in other applications. This latter result is most likely explained by partial occlusion of the cells by the vertical line formed by the nanobeads (Supplementary Figure S2). Indeed, traditional cell detection methods usually require a defined border between the cell and other cells or environmental elements. In this context, occlusion of cells by the beads disrupts the cell border within the image, as well as cell’s measured “circularity,” causing cells to erroneously be excluded from detection. Additionally, the beadline itself occasionally presents with morphological irregularities that may be detected as a cell, further obfuscating detection. We therefore sought to use machine learning to find a more reliable method to classify droplets based on the number of cells they contain.

**TABLE 1:** Classification accuracy of empty droplets, droplets containing a single cell and droplets containing multiple cells obtained with indicated conventional methods, in presence or absence of nanobeads in the droplets.

Image dataset	Detection Method	Classification accuracy (%)		
		Empty	Single cell	Multiple cell
Cells only	Hough transform	100	85	20
	MSER algorithm	99	21	26
	Segmentation algorithm	100	83	34
Cells with beads	Hough transform	99	31	4
	MSER algorithm	68	38	42
	Segmentation algorithm	99	62	42

The scores were calculated with 1,000 images for each condition.

**FIGURE 2:**
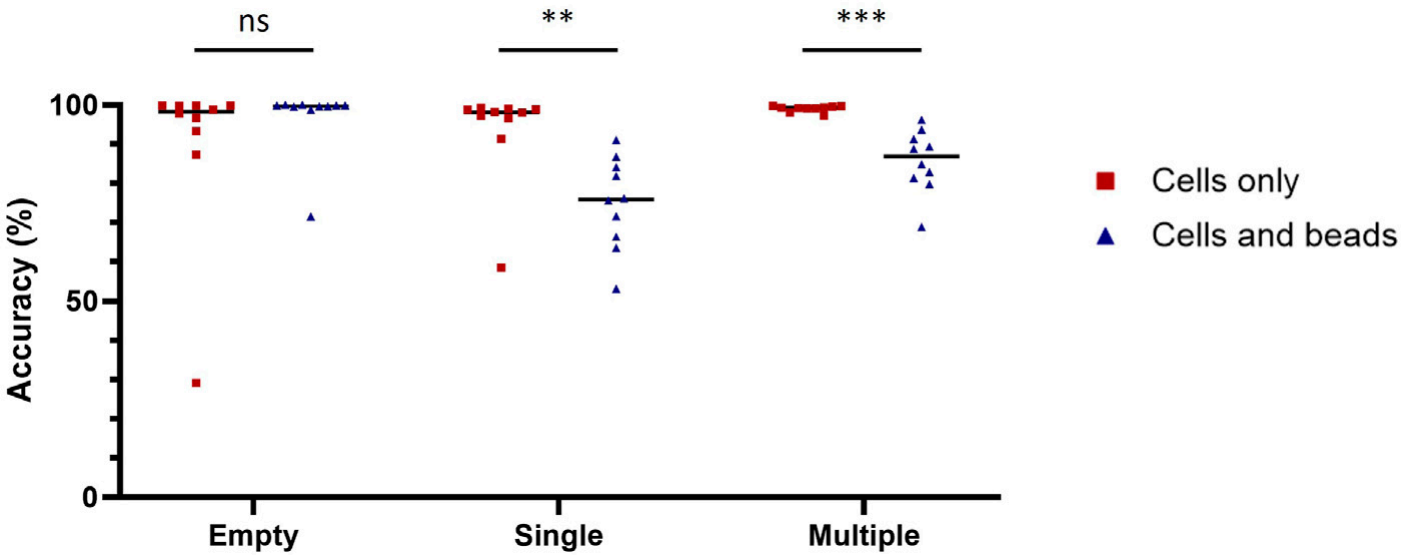
Diminishment of classification accuracy due to the beadline. Classification accuracy of model trained and tested with images of droplet containing cells only (red squares) or cells and beads (blue triangles) on empty droplets (Empty), droplets containing a single cell (Single) and droplets containing multiple cells (Multiple). The scores were calculated with 1,000 images for each condition. Scores were compared using Welch’s *t*-test (n = 10). **: *p* < 0.01; ***: *p* < 0.001; ns, not significant.

We chose ResNet-50, a 50-layer CNN architecture introduced in 2015, designed to address the problem of vanishing gradients in very deep neural networks (Song et al., 2018). ResNet-50 models are typically trained using a mini-batch stochastic gradient descent and these models have been shown to achieve state-of-the-art performance on a variety of domains including microfluidics (Gangadhar et al., 2023; Praljak et al., 2021; Sahaai et al., 2022). Before training the ResNet-50 neural network, we manually generated two training datasets (with or without nanobeads in the droplets) each containing three categories (empty, single-cell and multiple-cell). Each category was composed of 16,000 and 2,500 images for the datasets with and without beadlines, respectively. We then assessed the performances of ten model replicates for each dataset. Each replicate was tested against the images used to evaluate the accuracy of conventional methods. When trained and tested with droplets containing cells only, ResNet-50 reached accuracy scores above 99% for each category (Table 2). When trained and tested with droplets containing cells and beadlines, the accuracy for single-cell droplets dropped to 55%, whereas the accuracy for empty and multiple-cell droplets remained above 90%. Overall, ResNet-50 performances were significantly lower when classifying droplets containing cells and beads (Figure 2). The presence of a beadline within droplets therefore represented an obstacle to classification. The potential for nearby droplets to also introduce image distortion at the edges of the droplet image also likely affected the model.

**TABLE 2:** First and third quartiles of classification accuracy scores for empty droplets, droplets containing a single cell and droplets containing multiple cells obtained with ResNet-50 models (n = 10), in presence or absence of nanobeads in the droplets.

Training dataset	Testing dataset	First and third quartile of accuracy scores (%)		
		Empty	Single cell	Multiple cell
Cells only	Cells only	94.2–99.9	96.8–98.9	99.1–99.5
	Cells with beads	7.9–43.8	14.3–39.6	92.0–98.3
Cells with beads	Cells only	100–100	87.0–93.3	89.9–95.2
	Cells with beads	99.5–99.9	67.7–83.6	81.8–90.8

The scores were calculated with 1,000 images for each condition.

**FIGURE 3:**
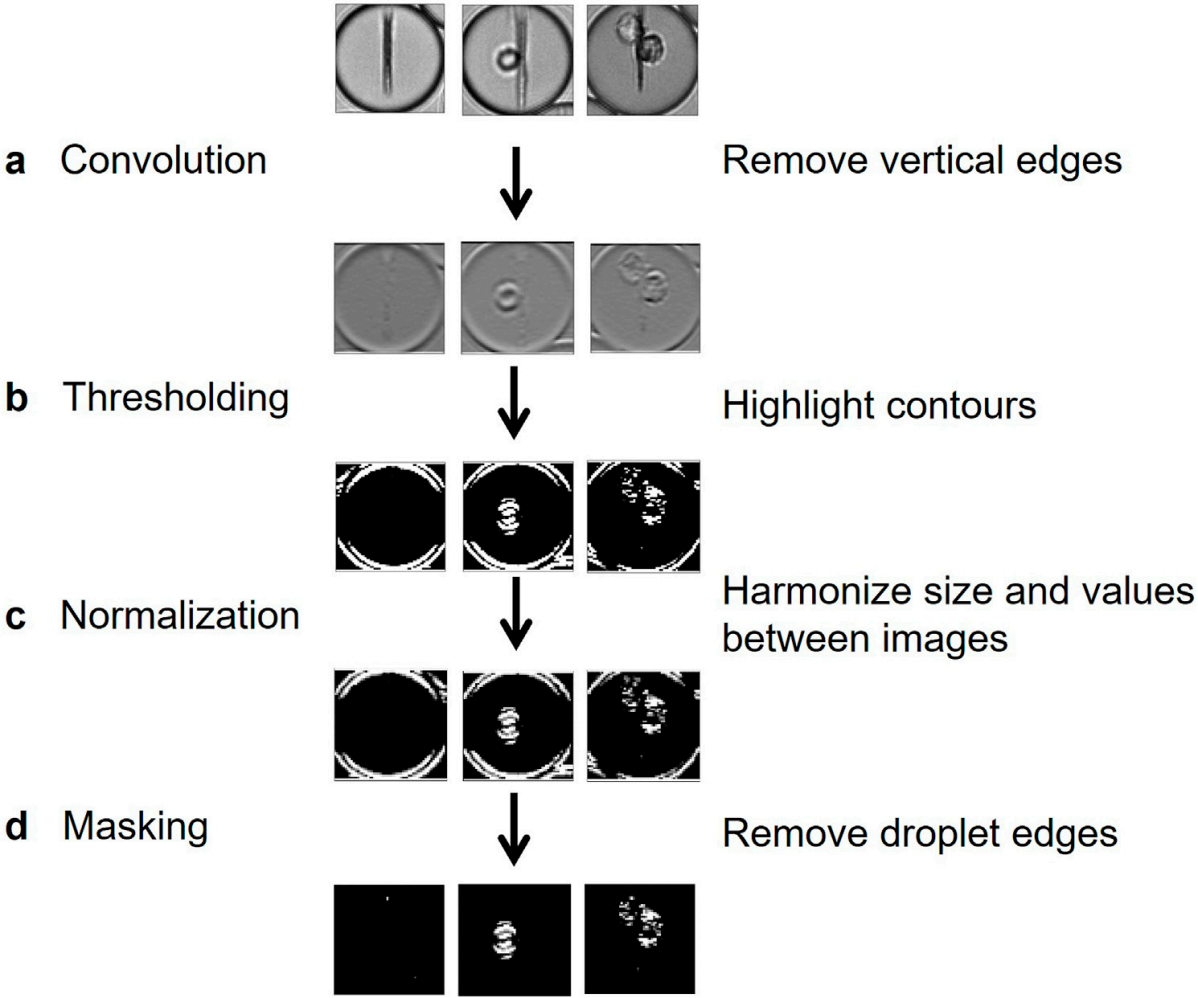
Flow chart of proposed preprocessing method. **(A)** A horizontal-edge-detecting convolution using Prewitt operator removes the beadline and other vertical structures. **(B)** A bisigmoidal threshold is applied to create firm segregation between the detected edges and the uniform grey. **(C)** Images are resized to create uniform input for model training. **(D)** The droplet edges are removed by applying a circular black mask, whose radius is slightly lower than the half-width of the image.

We therefore implemented a pre-processing method for removing information irrelevant to classification, such as the beadline and droplet borders, while also emphasizing meaningful information, which in this case represented the cells. Image preprocessing was composed here of four steps. First, a convolution using a Prewitt operator kernel (Baareh et al., 2018) was applied to remove the beadline and other vertical structures (Figure 3A). This caused the image background to be uniformly grey, whereas horizontal edges were either white or black; this difference is due to the asymmetry of the Prewitt operator. Then, a bisigmoidal filter was applied to enhance the contrast between the background and the contours (Figure 3B) (see Methods). All images were then resized, and pixel values were harmonized by applying an affine transformation (Figure 3C) (see Methods). Finally, droplet borders were removed by applying a circular binary mask, in which the central circle radius is several pixels less than that of the droplet (Figure 3D).

**FIGURE 4:**
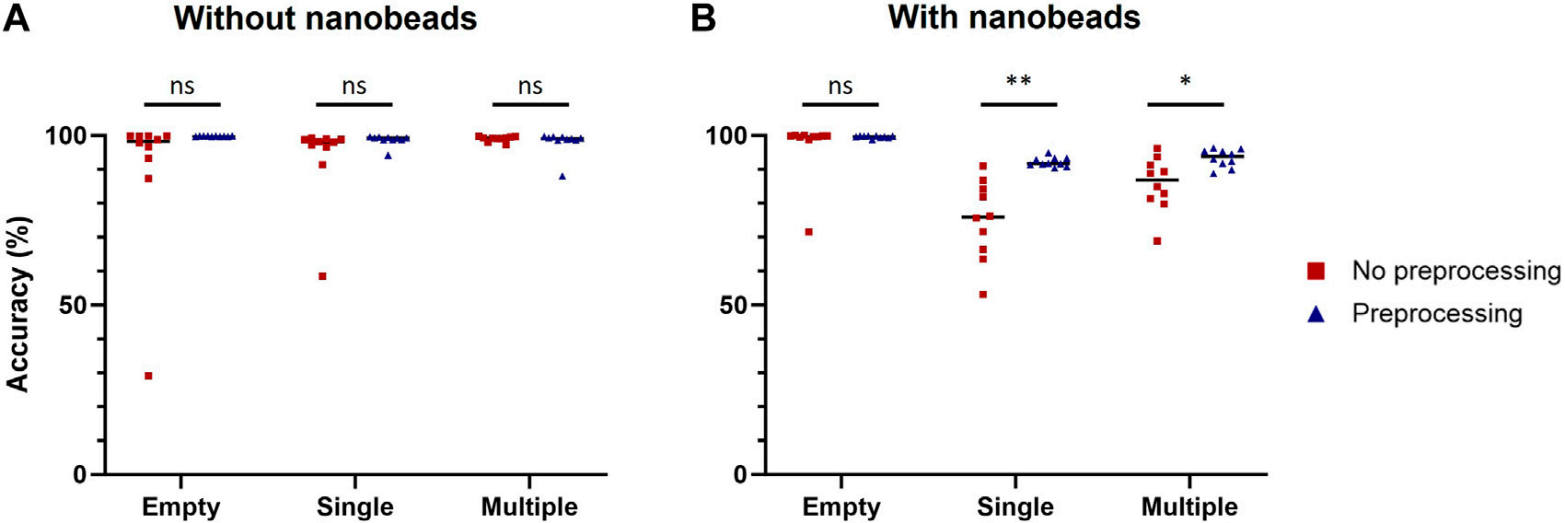
Improvement of classification accuracy due to preprocessing. Classification accuracy of models trained with raw or preprocessed images were assessed on empty droplets (Empty), droplets containing a single cell (Single) and droplets containing multiple cells (Multiple), without **(A)** or with **(B)** nanobeads. The scores were calculated with 1,000 images for each condition. Scores were compared using Welch’s *t*-test (n = 10). *: *p* < 0.05; **: *p* < 0.01; ns, not significant.

This preprocessing was then applied to each image of the training datasets used to compare computer-vision-based and machine-learning-based methods to build two new datasets of preprocessed images with cells only or with cells and nanobeads. Ten model replicates were trained for each dataset and their performances were assessed on the same validation datasets used for Table 2 (Figure 4). No statistically significant improvement due to the image preprocessing was observed when models were tested with images of droplets containing cells only. However, image preprocessing significantly improved prediction accuracy when the models were challenged with images of droplets containing cells and nanobeads. For single-cell droplets containing nanobeads, the median accuracy scores of models trained and tested with raw or preprocessed images were 75.9% and 91.7%, respectively. For multiple-cell droplets, the median accuracy scores of models trained and tested with raw or preprocessed images were 86.9% and 93.4%, respectively. These results demonstrate that the proposed image preprocessing improved prediction accuracy by approximately 20% and 7% in single or multiple-cell contexts, respectively. It is noteworthy that this preprocessing also significantly improved the confidence values associated with predictions (Supplementary Figure S3).

**FIGURE 5:**
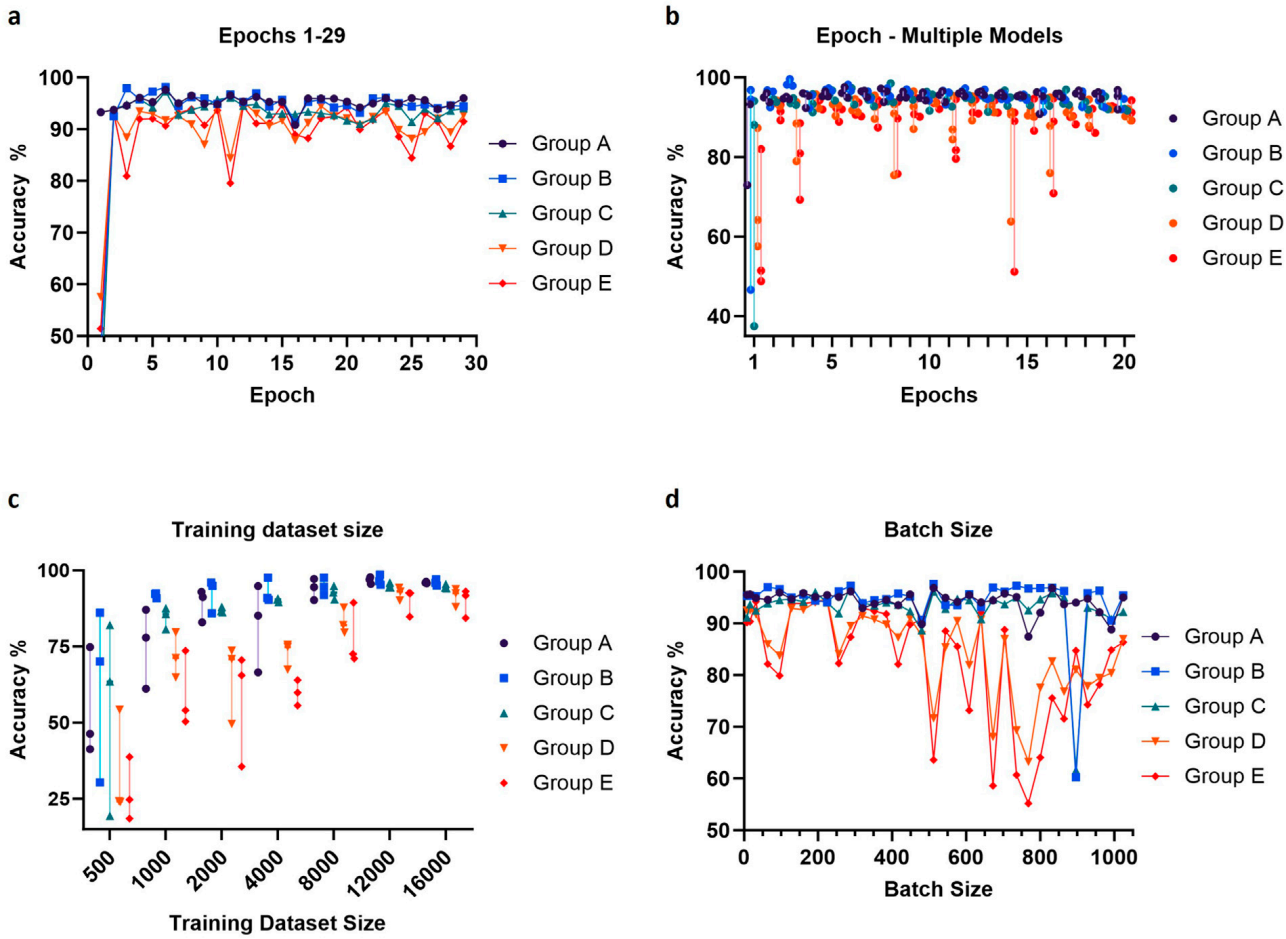
Adjustment of epochs number, training dataset size (per class) and batch size. The values of these parameters were optimized by evaluating validation accuracy using data from five independent experiments (Groups A-E). For each image, three hundred droplets were used to measure the accuracy. Prediction accuracy was calculated using either one **(A, D)** or three **(B, C)** trained models per parameter value. The default values for the parameters were training dataset size = 16,000 images per class, epochs number = 12 and batch size = 64.

We set out to optimize various training parameters, including the dataset size, the number of epochs, and the batch size. The training datasets again contained 3 classes of images, empty droplets, or no cells (empty), droplets with only a single cell (single), or droplets with multiple cells or clear aggregates (multiple). Accuracy was tested on droplet images across 5 independent experiments (300 images per experiment, 1,500 images in total) to investigate potential performance variability (Figure 5). As expected, the performance of the model improved as the training dataset increased, with the accuracy reaching a plateau around 12,000 images per class (Figure 5C). Concerning the number of epochs, an abrupt increase in accuracy was observed for the first several epochs, followed by a plateau and a trend to decrease after 10 epochs, corresponding to the onset of overfitting (Figures 5A, B). Large batch sizes led to irregular performances, as the model likely became unable to converge quickly enough; the optimal and most stable accuracies were obtained with batch sizes between 128 and 200 (Figure 5D). From these observations, we proceeded to train a final model using 16,000 images per class, and the gradient descent was performed using 6 epochs and a batch size of 160.

**FIGURE 6:**
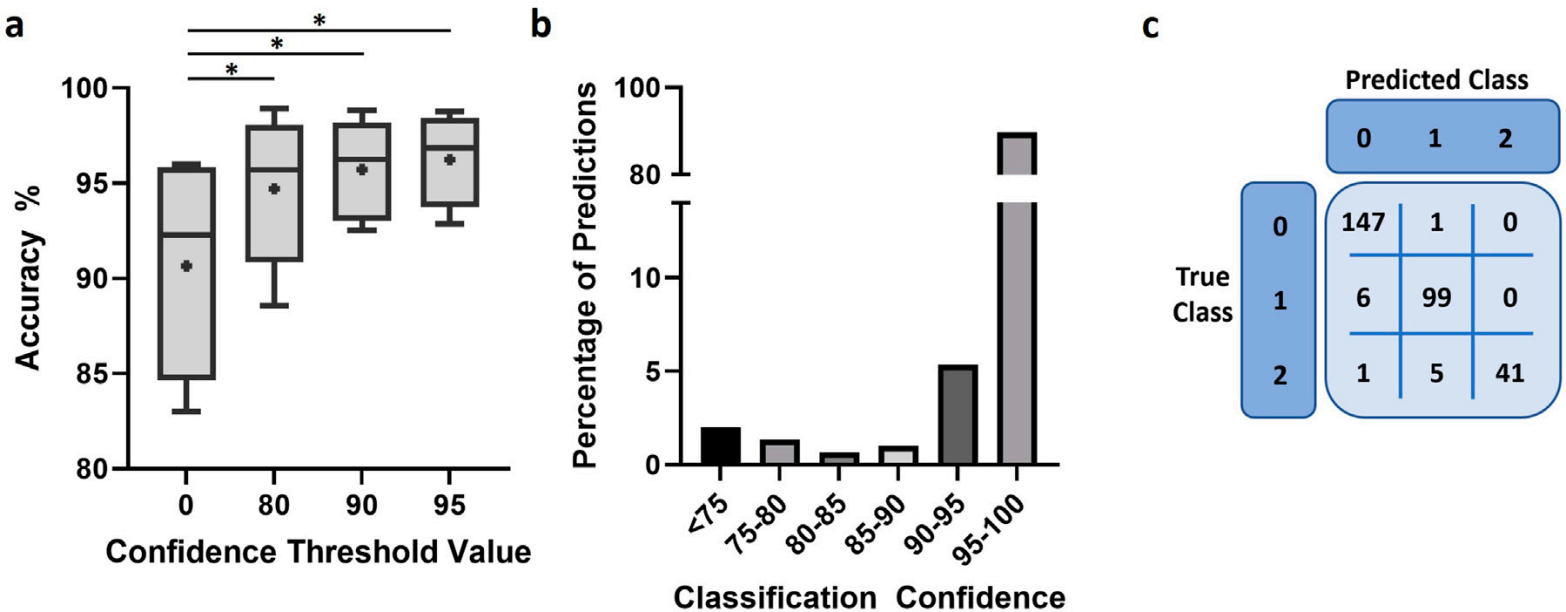
Model performance after training. **(A)** Accuracy of model predictions under different conditions of confidence restriction, n = 5 and “+” indicates mean. Scores were compared using paired *t*-test. *: *p* < 0.05 **(B)** Bar chart representing the proportion of predictions against the classification confidence of the trained model. **(C)** Representative confusion matrix used to assess model performance.

We used the same 5 independent experiments to further assess performance of the final model in representative conditions for applied experiment analysis. The model demonstrated a high degree of accuracy, over 90% in most images analyzed (Figure 6A). Moreover, imposing a confidence threshold to only include the most reliable predictions led to an increase of accuracy and reduced variability between experiments. This confidence threshold for classification exclusion did not result in notable amounts of data loss, 2% or 5% of all predictions when a threshold of 75 or 90 was applied, respectively (Figure 6B). We further investigated model performance through the confusion matrices of classifications to better understand the nature of errors made by the model (Figure 6C; Supplementary Figure S4). The model appeared minorly skewed towards under-detection of cells, with the number of cells in the droplet being underestimated when classification was incorrect.

## Discussion

In the process of optimizing and automating our existing DBMF data analyses, which had relied heavily on manual discrimination of droplet images, we faced limitations of classical image analysis approaches aimed at detecting cells in the absence of other structures that may hide part or the entirety of the cell. We explored the possibility of using a well-established machine learning classification approach based on the CNN ResNet-50, designed in 2015 to address the challenge of vanishing gradients in very deep neural networks, which can make it difficult for the network to learn and make accurate prediction (Song et al., 2018). ResNet-50 models have been shown to achieve state-of-the-art performance on a variety of domains including microfluidics (Gangadhar et al., 2023; Praljak et al., 2021; Sahaai et al., 2022). Although our large dataset could enable the training of a deeper model, we chose ResNet-50 rather than ResNet-102 or ResNet-152 as the variability and complexity of the features relevant for classification are relatively low. In this case a shallower model might be preferred to achieve a better generalization. Several studies have reported similar image classification accuracy scores between ResNet models (Bianco et al., 2018; Kumar et al., 2024; Sofian et al., 2018). Moreover, when an improved accuracy is reported for a deeper ResNet model (i.e., additional layers), it is to the detriment of analysis time (Bressem et al., 2020; Wightman et al., 2021). Even though we trained our model for the analysis of a stationary assay, the kind of pre-processing we present here could be adapted to other contexts involving live sorting. In such situations, classification speed would become a key parameter. We demonstrate high accuracy classification under our assay conditions where magnetic nanoparticles form a column (termed beadline) within the droplet that often occludes or mimics the shape of cells. Introducing an additional pre-processing module that was specific to the morphological properties of these images – the presence of vertically aligned linear structures – significantly improved the model training performance and the accuracy of the results.

In most known approaches to machine learning for image classification, minimal pre-processing is applied to the datasets or generic data normalization transformations are used with the aim of improving the numerical stability and the convergence time of the models (Shanker et al., 1996). Soldati, Ben, et al. (2018) previously applied CNNs to separate cell-containing droplets from empty droplets and debris-containing droplets. The assay they used in this study did not require non-cellular structures within the droplets. In a previous study, Anagnostidis et al. (2020) developed a CNN model to classify droplets containing both cells and non-cellular structures that rarely mask the cells, as they appear mostly transparent or are smaller than the cells. Hence, the latter model could reach accuracy scores of 80%–85% with minimally preprocessed images. Here we show that the presence of the beadline may represent a major obstacle to accurate classification, as shown by the decrease of accuracy to identify single-cell droplets (Table 2). In this scenario we show that adding an application-specific pre-processing step can be highly beneficial, guided by previous-knowledge-based assumptions on which image features do not carry any useful information for the classification. Thanks to this step, the model could reach accuracy scores above 90%. Also, the model we present here provides additional information as it can distinguish single-cell droplets from multiple-cell droplets. Other DBMF techniques that use non-cellular objects whose morphology is sufficiently different from cells (Ding et al., 2020) may benefit from adding a custom filter at the start of any image-based machine learning pipeline. To do so, our pipeline could be adapted by using a different kernel for the convolution step or by implementing additional filters based on morphological operations or other image treatment algorithms.

It is noteworthy that our full pipeline produced an improvement in results also for droplets that do not contain any beadline. In this case, the application of the circular mask before feeding the images in the neural network may have played a significant role in masking disturbances that could arise from surrounding droplets. This can be expected as CNNs alone are inherently local and insensitive to global (non-local) patterns (Song et al., 2018; Wang et al., 2018), and might advocate for the integration of non-local layers in networks used to analyze structured images. Our implementation of ResNet-50 for droplet classification offers a simple, robust solution for other investigators seeking to implement cell number classification into their DBMF analysis workflows.

We applied our approach to the classification of droplet images coming from a stationary experiment, in which droplets were immobilized in a horizontal-plane imaging chamber. However, the sole element needed to perform this classification is a bright-field image of the droplet. This classification method may also be applied to images from flowed DBMF systems, in which the images of single flowing droplets can be captured using a high-speed camera (Gérard et al., 2020). In such cases, the ability to quickly perform an automated classification could be used to directly trigger sorting decisions in real-time. Despite the availability of image flow cytometry and cell sorting, commercially available systems only allow for a limited set of information from the image to be used to gate and sort cells (Holzner et al., 2021); being able to feed the image through a pre-trained neural network and sort the droplets according to the output holds the potential of improving existing workflows in droplet-based assays where the recovery of rare or difficult-to-detect populations is required.

The application of deep learning methods to make “live” sorting decisions poses significative barriers, especially in fast high-throughput setups where all the computations must be done with strict and reproducible timing. With the computation setup we used (see Methods), our model is able to classify 1,000 droplets in less than 20 s, in other words with an average frequency of 50 Hz. Therefore, the model we described here could be adapted to a number of live sorting assay, as previous studies reported sorting frequency lower than 50 Hz (Beneyton et al., 2016; Anagnostidis et al., 2020). We believe that the increasing performance of currently available workstations and acquisition hardware, coupled with the smart design of synchronized acquisition setups will make this kind of model a valuable tool for an increasing number of DBMF assays, even based on higher sorting frequencies. Our work to implement machine learning for droplet cell number classification in a context with in-droplet structures to measure secretion significantly improves the throughput of such single cell assays as well as the scope of machine learning use for DBMF.

## Data Availability

The datasets presented in this study can be found in online repositories. The names of the repository/repositories and accession number(s) can be found below: Training and validation image datasets are available on Zenodo (Doi: 10.5281/zenodo.10810199). Scripts used in this work will be available as ESI.

## Supplementary material

SUPPLEMENTARY FIGURE 1 Illustration of computer-vision based methods to detect cells. Droplet images were classified using various methods to detect cells: Maximally Stable Extremal Regions (MSER) algorithm **(A)**, segmentation algorithm **(B)** or Hough transform **(C)**. The original images are in the left column. The detected signal is superimposed over the original image in the right column.

SUPPLEMENTARY FIGURE 2 Challenge posed by the presence of non-cellular structures to computer-based methods. Examples of incorrect cell detection due to non-cellular structures, using Maximally Stable Extremal Regions (MSER) algorithm **(A)**, segmentation algorithm **(B)** or Hough transform **(C)**. The original images are on the left. The detected signal is shown **(A)** on the right, or **(B, C)** superimposed over the original image in the middle and on the right.

SUPPLEMENTARY FIGURE 3 Improvement of classification confidence due to preprocessing. Classification confidence of models trained with raw or preprocessed images were assessed on empty droplets (Empty), droplets containing a single cell (Single) and droplets containing multiple cells (Multiple), without (left) or with (right) nanobeads. The scores were calculated with 1,000 images for each condition. Scores were compared using Welch’s t test (n = 10). * : *p* < 0.05; ns, not significant.

SUPPLEMENTARY FIGURE 4 Nature of errors made by the optimized model. The confusion matrices were established based on 300 images of 5 independent experiments (1,500 images in total). Each of these 5 panels represent an independent experiment.
