# Supplementary figures and images for: Deep learning enabled label-free microfluidic droplet classification for single cell functional assays
Source: Front Bioeng Biotechnol. 2024 Sep 18;12:1468738. doi: 10.3389/fbioe.2024.1468738 (PMC11445169; doi:10.3389/fbioe.2024.1468738)

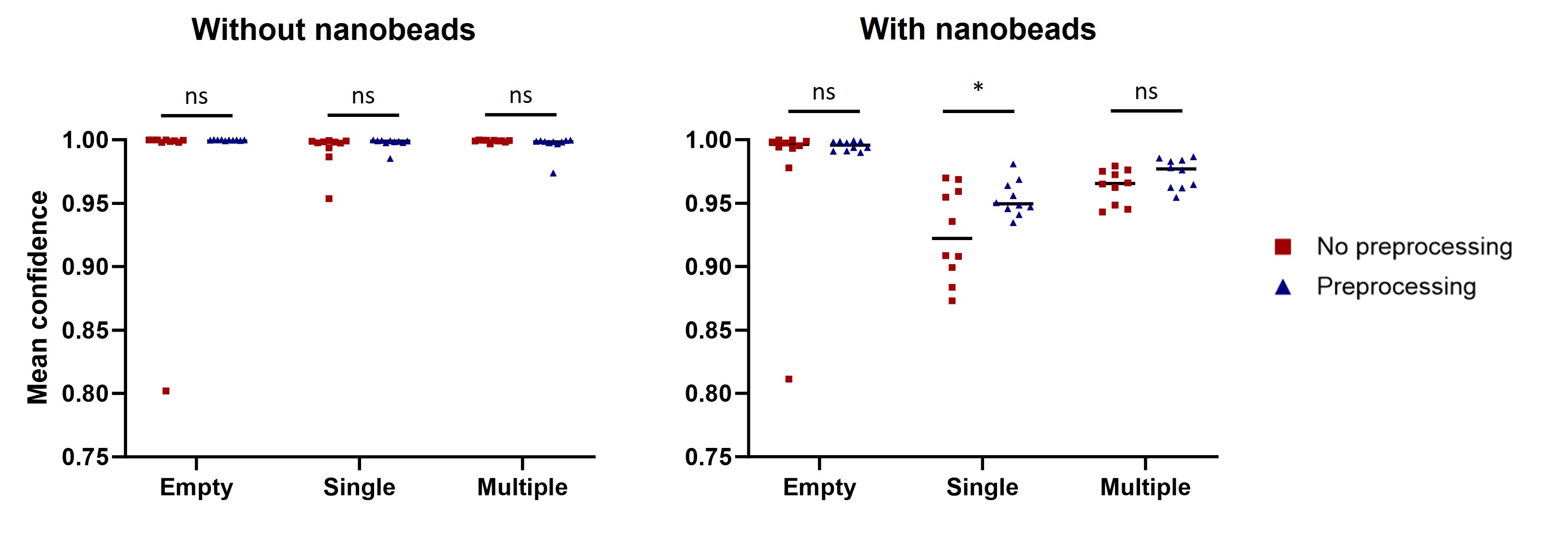

Supplement: Supplementary file 1 [file Image3.JPEG]

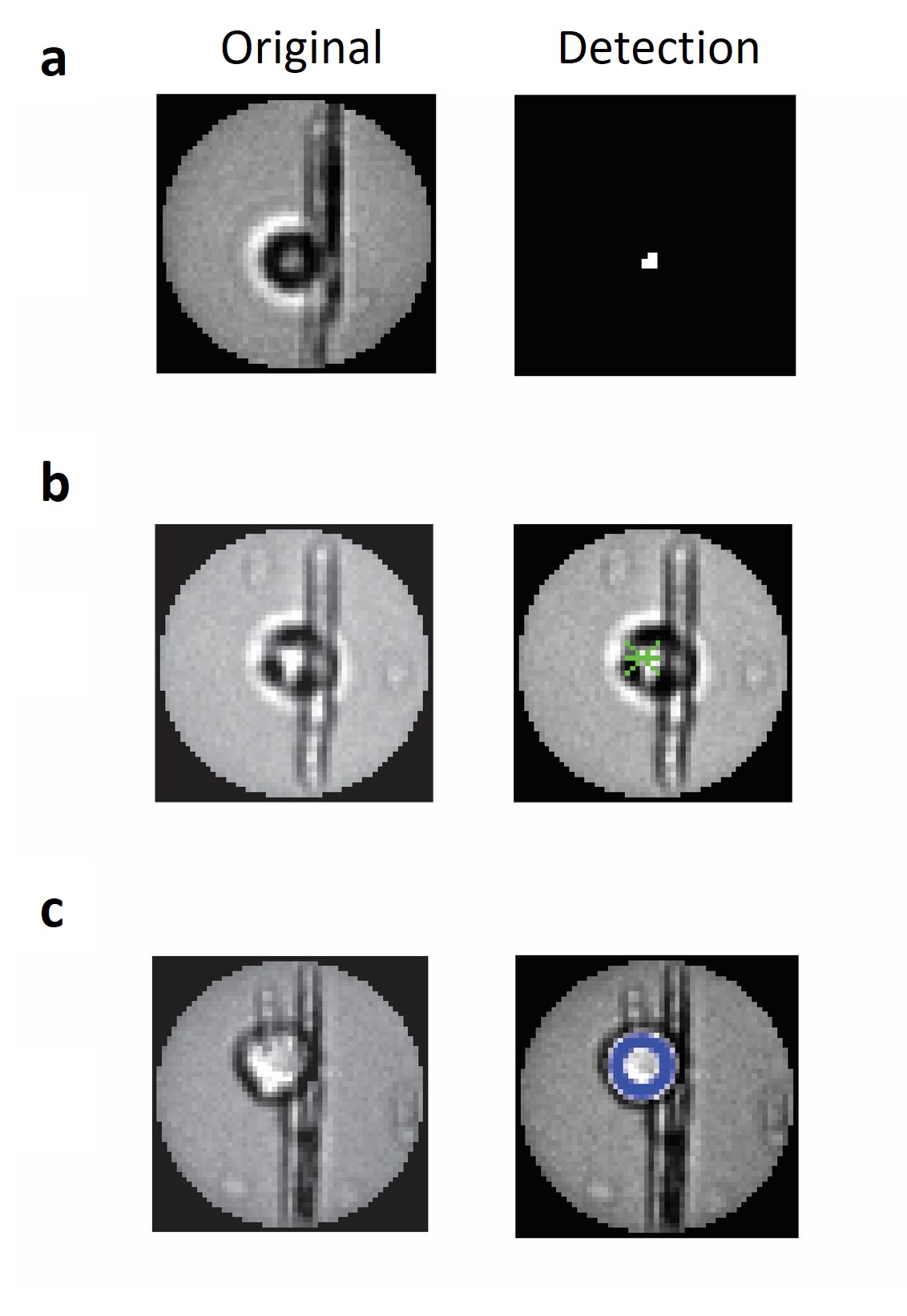

Supplement: Supplementary file 2 [file Image1.JPEG]

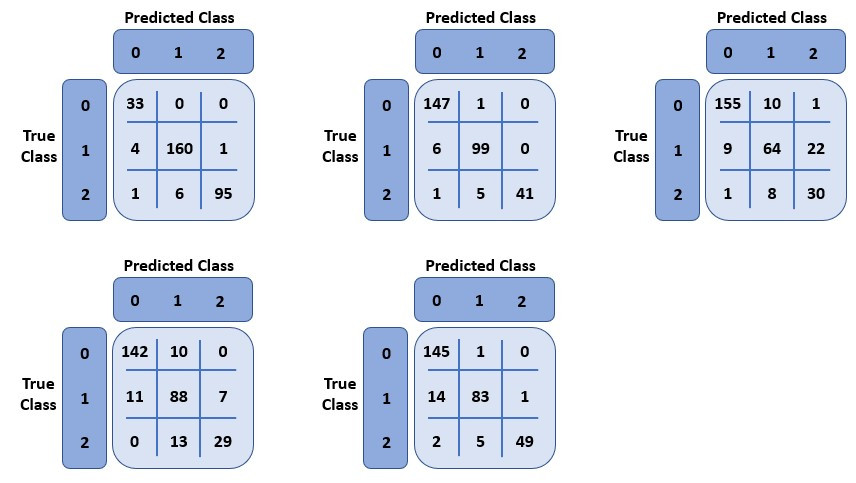

Supplement: Supplementary file 3 [file Image4.JPEG]

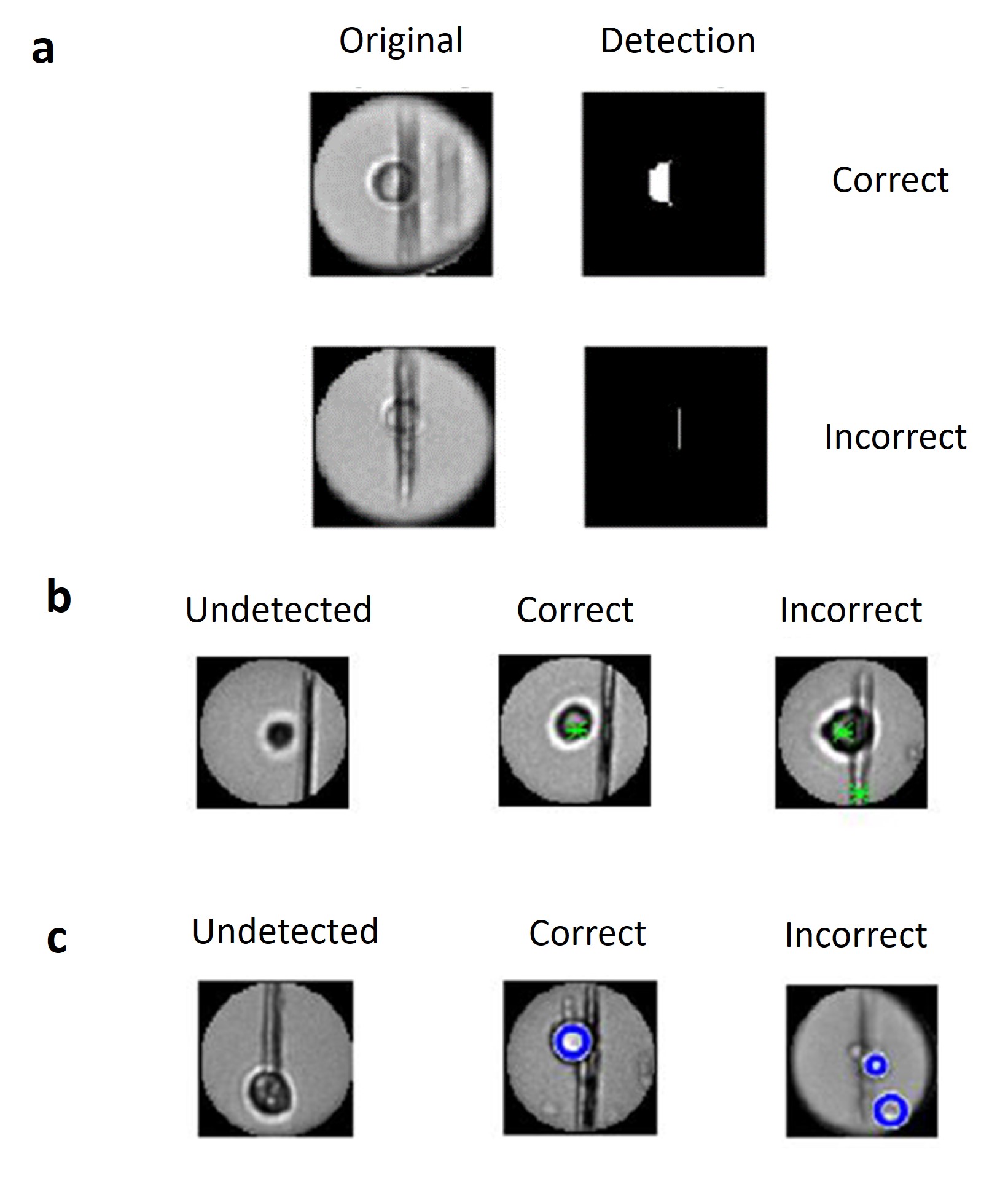

Supplement: Supplementary file 5 [file Image2.JPEG]
